# One-Pot Sonochemical Synthesis of ZnO Nanoparticles for Photocatalytic Applications, Modelling and Optimization

**DOI:** 10.3390/ma13010014

**Published:** 2019-12-18

**Authors:** Muhammad Tayyab Noman, Michal Petru, Jiří Militký, Musaddaq Azeem, Muhammad Azeem Ashraf

**Affiliations:** 1 Department of Machinery Construction, Institute for Nanomaterials, Advanced Technologies and Innovation, Studentská 1402/2, Technical University of Liberec, 461 17 Liberec, Czech Republic; michal.petru@tul.cz; 2 Department of Material Engineering, Faculty of Textile Engineering, Studentská 1402/2, Technical University of Liberec, 461 17 Liberec, Czech Republic; jiri.militky@tul.cz (J.M.); musaddaqazeem@yahoo.com (M.A.); 3 Department of Fibre and Textile Technology, University of Agriculture, Faisalabad 38000, Pakistan; azeemashraf786@hotmail.com

**Keywords:** ZnO, ultrasonic, nanoparticles, photocatalysis, optimisation

## Abstract

This present study proposed a successful one pot synthesis of zinc oxide nanoparticles (ZnO NPs) and their optimisation for photocatalytic applications. Zinc chloride (ZnCl_2_) and sodium hydroxide (NaOH) were selected as chemical reagents for the proposed study. The design of this experiment was based on the reagents’ amounts and the ultrasonic irradiations’ time. The results regarding scanning electron microscopy (SEM), X-ray diffraction (XRD) and Raman spectroscopy confirmed the presence of ZnO NPs with pure hexagonal wurtzite crystalline structure in all synthesised samples. Photocatalytic activity of the developed samples was evaluated against methylene blue dye solution. The rapid removal of methylene blue dye indicated the higher photocatalytic activity of the developed samples than untreated samples. Moreover, central composite design was utilised for statistical analysis regarding the obtained results. A mathematical model for the optimisation of input conditions was designed to predict the results at any given point. The role of crystallisation on the photocatalytic performance of developed samples was discussed in detail in this novel study.

## 1. Introduction

During the past few years, the research on metal oxides, particularly zinc oxide (ZnO), has remarkably increased and gained enormous interest from researchers in chemistry, materials science, physics, medical, textiles and many other fields of science [1,2,3,4,5,6,7,8,9,10,11,12]. ZnO belongs to a family of metal oxide semiconductors. ZnO is an n-type semiconductor with wide band gap (3.37 eV) and having large exciton binding energy (60 meV). On the nanoscale, ZnO shows exceptional physicochemical properties that are beneficial in many industrial applications. Many researchers have successfully used ZnO nanostructures in photocatalytic [13,14,15,16], photovoltaic [17,18,19,20], biomedical [21,22,23,24] and sensing [25,26,27,28,29,30,31] applications, as it provides large surface area as compared to its bulk counterpart. The demand of highly efficient, durable and robust photocatalysts for wastewater treatment makes ZnO nanostructures a reliable candidate for photocatalytic applications.

Researchers have been concluded that the photocatalytic performance of nanomaterials significantly depends upon the synthesis route, size, shape, crystallinity and dimension [32,33,34,35,36]. At present, different researchers have prepared ZnO nanostructures by different synthesis methods i.e., sol–gel [37,38,39], hydrothermal [40,41,42], co-precipitation [43,44,45], green [46,47,48,49], electrospinning [50,51] and sonochemical [52,53]. The sonochemical method among all mentioned methods has been proven to be a more economic, efficient and facile approach used for the synthesis of nanomaterials. This sonochemical method works on the principle of acoustic cavitation. In liquids, ultrasonic energy induces physicochemical changes in a material through low-pressure/high-pressure waves. These waves lead to create a huge number of unstable vacuum bubbles that aggressively collide with each other due to pressure difference, and develop extremely local conditions, i.e., local pressure and temperature raised up to 20 MPa and 5000 K, respectively, with cooling rate 10^10^ K·s^−1^ [54]. The size and crystallinity of the synthesised structures have been very well controlled by the sonochemical method, and that is another advantage of using this method.

In an experimental study, Pholnak et al. used an ultrasonic homogeniser as a tool to fabricate ZnO NPs under varying shapes i.e., sphere, cube and cylinder. They sonochemically treated zinc nitrate with hexamethylenetetramine at ambient temperature. They reported that the ultrasonic homogeniser is a better tool for nanomaterials’ synthesis than the ultrasonic bath and sonoreactor. Moreover, scanning electron microscopy (SEM) and X-ray diffraction (XRD) results confirmed the formation of ZnO NPs with an average particle size of 70 nm [55]. In another study, Hipolito and Martinez used the sonochemcial method and synthesised ZnO NPs at room temperature and further used them as a photocatalyst in hydrogen gas generation. They reported that the high surface area and smaller particle size of ZnO NPs significantly enhances hydrogen production. Furthermore, they suggested ultrasonic energy an efficient and cost-saving tool, as they used the ultrasonic method for avoiding long reaction time and high temperature [56]. Ma et al. fabricated two-dimensional (2D), hierarchical, fern-like nanoleaves of ZnO in water at room temperature with significantly enhanced photocatalytic performance by this ultrasonic method. The results obtained by different characterisation techniques i.e., SEM, XRD and Transmission electron microscopy (TEM), which confirmed the synthesis of 2D ZnO through the ultrasonic method. They concluded the ultrasonic method as a clean and economic route for large scale production of novel nanostructures [57]. Mahmoodi et al. worked on the modelling of the photocatalytic dye removal of basic blue 41 and basic red 46 by using ZnO NPs, and explained that under optimised conditions the maximum dye removal achieved by ZnO was 72.56% [58]. Dhiman et al. studied the modelling and optimisation of the dye removal efficiency of ZnO NPs by using acridine orange. They used the green chemical method for the synthesis of ZnO NPs and reported a maximum of 72% dye removal efficiency for the synthesised pure ZnO [59]. In another modelling and optimisation study of ZnO, the photocatalytic degradation of RB 19 and RB 21 was investigated by Rodrigues et al. under a UV photoreactor. They obtained 100% dye removal for RB 19, but the time taken for this was more than six hours [60].

In this novel study, ultrasonic homogeniser was used for one pot sonochemical synthesis of ZnO NPs for photocatalytic degradation of methylene blue. This study was conducted to investigate the influence of ultrasonic energy onto the structure and morphology of ZnO NPs, as well as the synergistic role of ZnO NPs onto photocatalytic applications. The process variables i.e., amount of zinc chloride (ZnCl_2_) and sodium hydroxide (NaOH), and the total time for ultrasonic irradiations, were adjusted by central composite design to attain the optimum conditions. To the best of our knowledge, there is no literature available regarding the synthesis of ZnO in this manner. This work represents a unique demonstration about the modelling and optimisation of as synthesised ZnO. Moreover, regarding the reagents used in this experimental study, it is supposed that this method is a robust one that could be extended for other zinc precursors and synthesis methods.

## 2. Materials and Methods

### 2.1. Materials

Zinc chloride (ZnCl_2_), sodium hydroxide (NaOH), ethanol (C_2_H_5_OH) and methylene blue (MB) dye with the chemical formula C_16_H_18_ClN_3_S were received from Sigma-Aldrich (Prague, Czech Republic). The chemicals were used as received.

### 2.2. Design of Experiment

**Figure 1:**
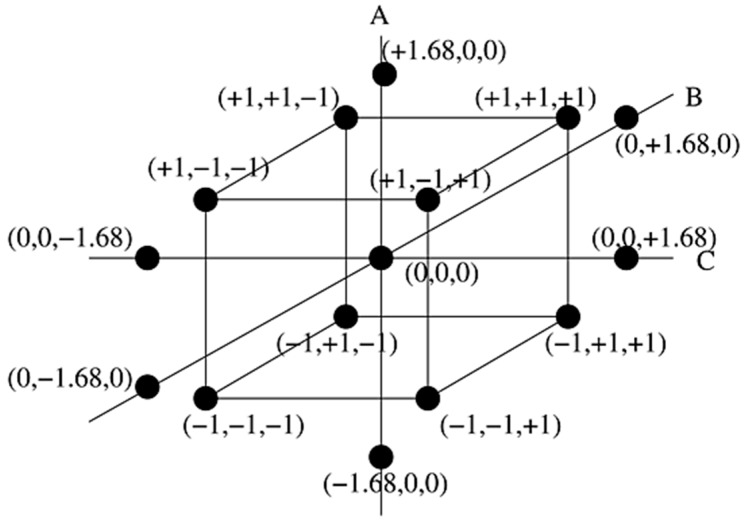
General form of three factors Central Composite Design (CCD) with coded values.

A Central Composite Design (CCD) is a set of experimental designs with three different design points i.e., factorial points (±1), a centre point (0) and star/axial points (±α). For a three factors CCD, the value of α is 1.68. The general form of a CCD with three input variables/factors (A, B, C) and their coded values (±1), centre point (0) and axial/star points (±α) is illustrated in Figure 1. The CCD is a valuable asset in determining the response surfaces by fitting a quadratic model in order to estimate the effect of curvature, or to find out the maxima or minima of a variable. Table 1 illustrates the input variables (factors) and the factors level setting in their coded form based on CCD.

The design of experiment (DOE) with different amounts of ZnCl_2_ and NaOH under varying sonication time based on CCD with experimental results is illustrated in Table 2. Some preliminary experiments were conducted to calculate the optimal point of each variable before starting the final experiment. The influence of variables on results was adjusted by using quadratic Equation (1).

(1) $$Y=b_{0}+\sum^{\;}b_{i}X_{i}+\sum^{\;}b_{i.j}X_{i}X_{j}+\sum^{\;}b_{i.i}X_{i}^{2}\;i\geq j\;i,j=1,2,3$$

where *b*_0_ is the coefficient of constant term, *b_i_* represents the coefficient of linear term, *b_i.j_* represents the coefficient of two factors interaction, and *b_i.i_* is the coefficient of quadratic term, respectively [54].

### 2.3. Synthesis of ZnO NPs

**Figure 2:**
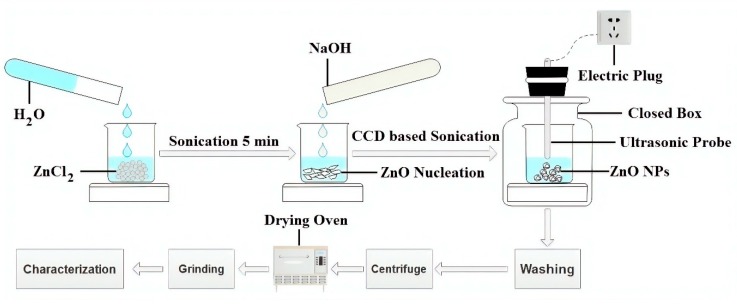
Graphical representation of sonochemical synthesis of ZnO NPs.

In one pot synthesis of zinc oxide nanoparticles (ZnO NPs), varying amounts of ZnCl_2_ (1.5 g to 18.5 g) were added into a beaker and then we adjusted the amount of water so that the total volume of the solution was maintained at 100 mL. During all experiments, distilled water was utilised. Sonicate the solution for 5 min to make a homogenous solution. A varied amount of NaOH granules (3.3 g to 11.7 g) was added into the running solution. To complete the reaction mechanism, the solution was then sonicated for different time intervals (19.7 min to 70.2 min) based on CCD under ultrasonic probe homogeniser (Bandelin Sonopuls HD 3200, Bandelin Electronic GmbH & Co. KG, Berlin, Germany, 20 kH_Z_, 200 W, 50% efficiency). The effective power of ultrasonic waves emitted into the solution was 100 Wcm^−2^, which value was experimentally determined by calorimetric measurement. After the completion of the sonication process, the resulting white flocculates were washed five times with ethanol to remove the excess amount of trashes and impurities. Afterwards, the white flocculates were centrifuged at 4000 rpm for 15 min to separate solid particles from liquid. The centrifuged solid was dried at 80 °C for 1 h in an oven to remove the remaining percentage of moisture and organic impurities. The centrifuged solid was then grinded to obtain the fine powder of ZnO that further characterised it. During our experiments, we observed that Sample 3 provided the maximum MB removal (%). So, in order to compare and to investigate the essential impact of the ultrasonic method, a similar sample was synthesised by the conventional magnetic stirring method under same experimental conditions without sonication, and named as sample M, as provided in Table 2. The schematic illustration of the experimental setup is given in Figure 2.

### 2.4. Characterisation of ZnO NPs

The surface topography and morphological changes of sonosynthesised ZnO NPs were observed by Ultra High-Resolution Scanning Electron Microscope UHR-SEM Zeiss Ultra Plus (Carl Zeiss Meditec AG, Jena, Germany) with an accelerating voltage of 2 kV. The charging effect was eliminated by the use of a charge compensator (local N_2_ injection). For the investigation of crystal structure, XRD patterns were collected by an X’Pert PRO X-ray diffractometer (Malvern Panalytical Ltd., Malvern, UK) using Cu Kα radiation of wavelength λ = 0.15406 nm with a scanning angle (2θ) range 5–80° with step size of 0.02° at voltage and current of 40 kV and 30 mA, respectively. The collected patterns were compared with standard patterns of the International Centre for Diffraction Data (ICDD) Powder Diffraction File (PDF: 89-7102) and further analysed. Moreover, Raman spectroscopy (Thermo scientific DXR Raman spectroscope, Thermo Fisher Scientific, Waltham, MA, USA) was utilised to detect the purity of the crystal phase. In order to calculate the size of the crystals, Scherrer’s crystallite Equation was used as given below:

(2) $$D=\frac{K\lambda }{\beta Cos\theta }$$

In Equation (2), *D* represents the crystallite size calculated through line broadening of plane reflection whereas, *λ* represents the wavelength of the X-ray radiations. *β* is the full line width at half-maximum height (FWHM) and *K* represents the shape constant with a constant value i.e., 0.89.

### 2.5. Photocatalytic Activity of ZnO NPs

Photocatalytic activity of developed samples of ZnO NPs was investigated by photodegradation of MB dye solution under UV light irradiations. For this study, 0.01% (*w*/*v*) solution of MB dye was prepared, and 0.5 g·L^−1^ of the as synthesised nano ZnO (Sample 3 and Sample M) was mixed in running solution. The suspension was kept in the dark for 40 min to reach equilibrium. The suspension was then exposed to a 500 W xenon lamp for 2 h. The distance between the UV lamp and the suspension was 30 cm, and the intensity of the UV light was 20 W·m^−2^, depending on the distance between the lamp and the sample. After a certain time, an aliquot was taken out and a UV-vis spectrum was recorded on a UV-1600PC Spectrophotometer (VWR International, Radnor, PA, USA). The characteristic concentration peak of MB was obtained at 668 nm of wavelength. The colour removal percentage (*CR*%) was calculated by the given equation:

(3) $$CR\%=\left[1-\frac{C}{C_{0}}\right]\times 100$$

In Equation (3), *C*_0_ and *C* represents initial and final concentration of MB dye in the solution, respectively.

## 3. Results and Discussion

### 3.1. Characterisation of ZnO NPs

**Figure 3:**
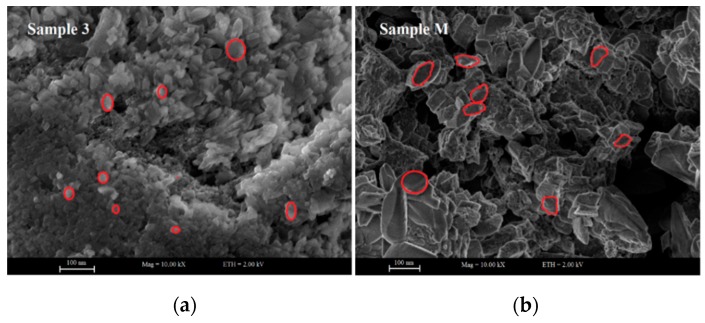
Scanning electron microscopy (SEM) analysis for (**a**) Sample 3 developed with optimal conditions Zinc chloride (ZnCl_2_) 15 g, sodium hydroxide (NaOH) 10 g, Sonication time 60 min, (**b**) Sample M developed without sonication.

The results regarding the surface topography and morphology of synthesised ZnO NPs were collected by SEM micrographs and illustrated in Figure 3. SEM results explained that sonication induced a great impact on the particle size of the resulting ZnO NPs. We observed that crystallite size for Sample 3 is two times smaller than Sample M. For Sample 3, we observed a homogenous distribution and quasi-spherical morphology of ZnO NPs, while an elliptical shape with sharp edges was observed for Sample M. The average particle size for Sample 3 and Sample M was 28 nm and 70 nm, respectively, as calculated from SEM images by ImageJ software (version 1.52p). These results are in good agreement with the findings of our previous investigation in which we concluded that ultrasonic irradiations play a significant role onto the smaller size and morphology of metal oxide nanoparticles [61].

**Figure 4:**
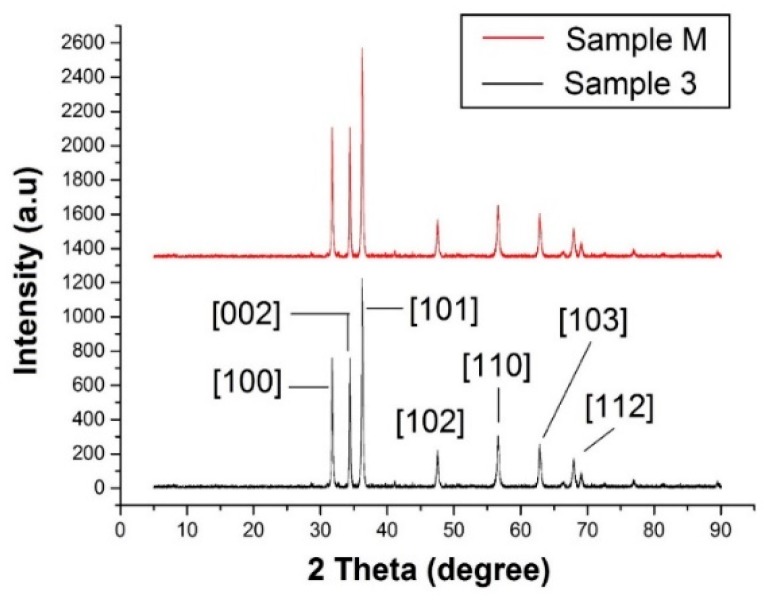
XRD pattern for Sample 3 developed with optimal conditions ZnCl_2_ 15 g, NaOH 10 g, Sonication time 60 min, and Sample M developed without sonication.

XRD analysis is a valuable asset in order to determine the crystal structure and crystallite size of a samples. The XRD patterns for observed samples (Sample 3 and Sample M) showed that the synthesised ZnO NPs possessed a pure hexagonal wurtzite crystal structure, as all the obtained peaks under XRD analysis for both methods (sonochemical and conventional stirring) matched with the ICDD file (PDF: 89-7102). In Figure 4, the highest peak obtained at 2θ = 36.2° is the characteristic crystalline peak of hexagonal wurtzite crystals of pure ZnO that follows [101] plane reflection. Moreover, a series of crystalline peaks at 2θ = 31.7°, 34.4°, 47.5°, 56.6°, 62.8° and 67.9° follow the [100], [002], [102], [110], [103] and [112] planes, respectively. We observed that hexagonal wurtzite crystals of nano ZnO were formed by both methods with a significant difference regarding crystal size for these methods. The average particle size for observed samples calculated by Scherrer’s Equation was 28.1 nm and 70.8 nm for Sample 3 and Sample M, respectively. The average particle size of all the samples prepared by the sonochemical method was 28 nm. The samples prepared by this sonochemical method have three times less particle size than the conventional method, which explains the significant role of the ultrasonic method in the synthesis mechanism of nanomaterials. The XRD results for particle size are in good agreement with the SEM results. Furthermore, no other phase (impurities) i.e., Zn(OH)_2_, was found during the XRD analysis.

**Figure 5:**
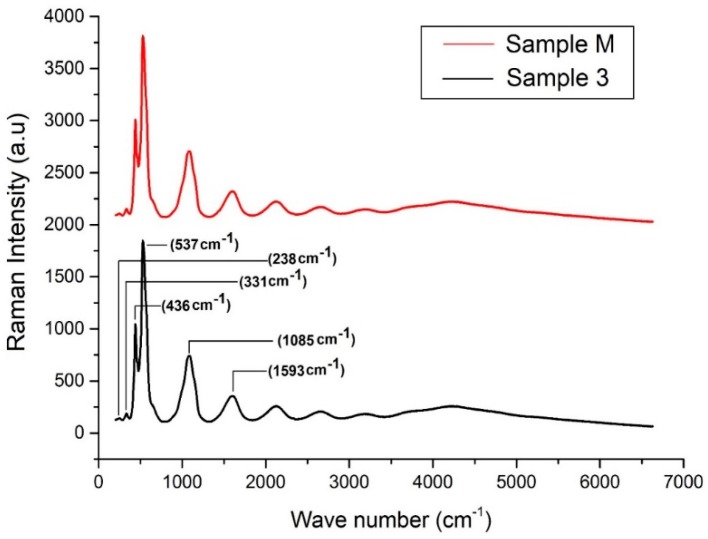
Raman spectrum for Sample 3 developed with optimal conditions ZnCl_2_ 15 g, NaOH 10 g, Sonication time 60 min, and Sample M developed without sonication.

Raman analysis is one of the finest modes for identifying the purity of a crystal lattice or the phase purity of a molecule. The results regarding the Raman analysis for all prepared samples are presented in Figure 5. It was observed that all the Raman bands matched with the characteristic peaks of pure hexagonal wurtzite ZnO. Factor group analysis for vibrational modes of crystals explained that the pure wurtzite crystal phase of ZnO consists of given Raman active modes i.e., (238, 331, 436, 537, 1085 and 1593 cm^−1^). In Figure 5, a strong peak at 537 cm^−1^ was observed, which is the metaphor for wurtzite ZnO NPs. Moreover, no characteristic peak of impurity i.e., Zn(OH)_2_, was found during analysis. The Raman results are significant and consistent with XRD results, and both techniques confirmed the formation of pure hexagonal wurtzite crystal of nano ZnO.

Specific surface area and pore size distribution are significantly influential microstructural properties of ZnO NPs highly dependent upon the geometry, morphology and porosity of as synthesised NPs. These parameters were measured by the Brunauer–Emmet–Teller (BET) method under N_2_ (−196 °C) atmosphere. The volume of a gas adsorbed is considered as the total area including the specific surface area and pore size. The average specific surface area of ultrasonically prepared samples (Sample 3) was 107 m^2^·g^−1^. The experimentally-determined values of surface area, pore volume and pore size distribution of all samples are provided in Table 3.

### 3.2. Photocatalytic Activity of ZnO NPs

**Figure 6:**
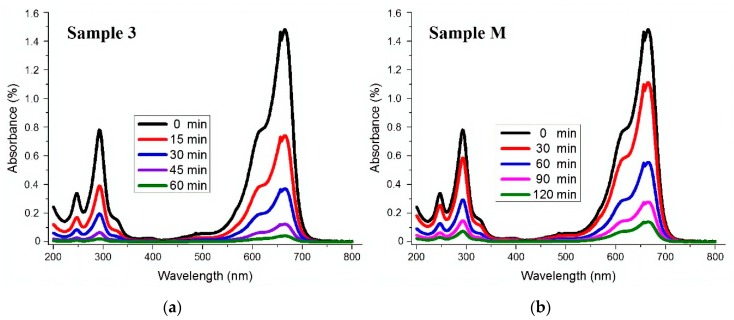
UV-Vis spectral changes as a function of irradiations time in MB dye solution containing synthesised ZnO (**a**) Sample 3 developed with optimal conditions ZnCl_2_ 15 g, NaOH 10 g, Sonication time 60 min, (**b**) Sample M developed without sonication.

The photocatalytic activity of as synthesised ZnO NPs was evaluated against MB dye solution. 0.5 g·L^−1^ of the prepared ZnO NPs was used for 50 mg·L^−1^ of MB dye. It was observed that under UV light irradiations, samples prepared by the ultrasonic method completely degraded MB dye in less than 60 min, while for Sample M, the degradation took a longer time. The results are quite obvious, as we obtained a smaller particle size by the sonochemical method than any conventional method. The results of MB degradation are in good agreement with BET results. The results of MB degradation for Sample 3 and Sample M are illustrated in Figure 6. In order to confirm that MB degradation was only due to ZnO NPs, a controlled sample of MB dye (without ZnO NPs) was exposed to UV light. This sample did not change its colour even after a longer time. This confirmed that MB degradation was only due to the presence of ZnO NPs in the dye solution. The overall results explain that sonochemically-prepared samples showed higher photocatalytic performance for MB than Sample M. UV-vis spectral changes in the MB dye solution as a function of UV light irradiations’ times are illustrated in Figure 6.

**Figure 7:**
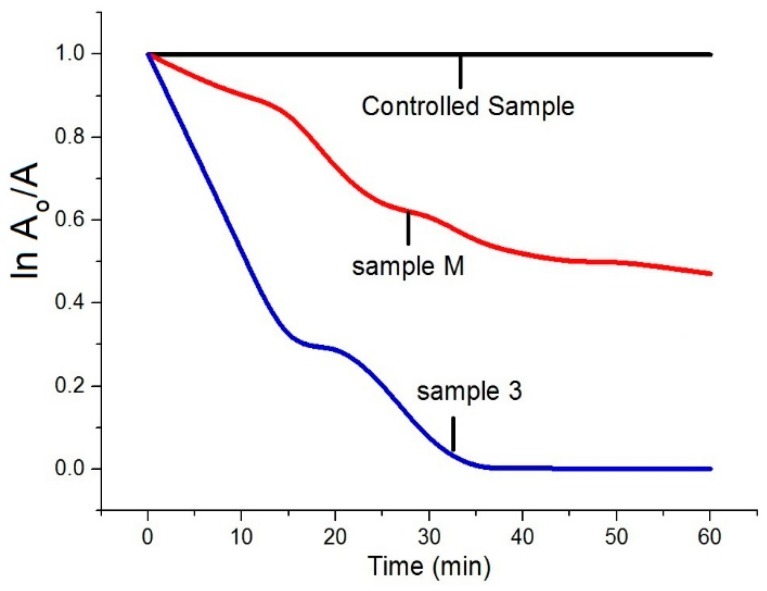
Time-dependent photocatalytic removal of MB. A_o_ show the absorbance at time t = 0, whereas A represents the absorbance at time t = ∞, respectively.

**Figure 8:**
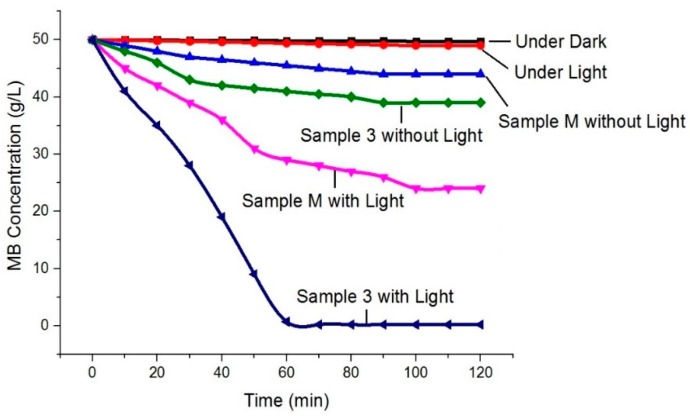
Behaviour of MB degradation under different conditions.

The ln A_o_/A plot for the complete degradation of MB against irradiation time is illustrated in Figure 7, where A_o_ represents the absorbance at time t = 0 min, and A represents the absorbance after a complete MB degradation at time t = ∞ min. The results explained that in a controlled sample (without ZnO NPs), no change was observed in MB degradation even after longer time, while 53% change was observed for Sample M as it is synthesised by a conventional method. However, a complete 99.9% MB degradation was observed in case of Sample 3. Furthermore, in order to investigate the behaviour of MB under different conditions, i.e., in the presence of dark, in the presence of light, in the presence of Sample M (with and without light) and in the presence of Sample 3 (with and without light), had also been evaluated, and the results are illustrated in Figure 8. Under dark conditions without ZnO NPs, the MB colour remained unchanged. Under irradiations (light conditions) without ZnO NPs, we still did not observe any significant change. For Sample M, 7% and 48% degradation of MB dye was observed under dark and light conditions, respectively. 16% colour change was observed for Sample 3 under dark conditions, whereas a significant colour change of 99.9% was observed for Sample 3 during light irradiations that showed the excellent photocatalytic activity of prepared ZnO NPs. It was noted that the colour change of MB under dark conditions for both samples (Sample M and Sample 3) was only because of the extra white colour of the prepared samples, which means that even with the photocatalyst, the process of photocatalysis was not started without light. So, we concluded that for photocatalytic degradation of pollutants, the presence of both, i.e., light and photocatalyst, is necessary. The influence of the ultrasonic method was significantly high, as we observed 99.9% MB degradation for Sample 3.

**Figure 9:**
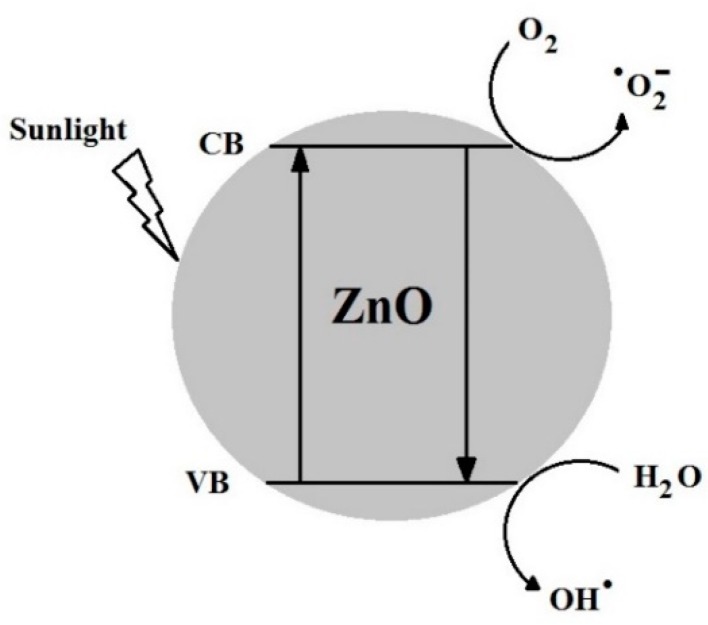
Proposed reaction mechanism on the surface of ZnO NPs for the generation of a reactive oxygen species (ROS).

Photocatalysis is a dynamic mechanism and the most fundamental property of nano ZnO that triggers a series of oxidation and reduction reactions. In photocatalysis, nano ZnO absorbs light energy and breaks down the long chain organic molecules (pollutants) into smaller fragments i.e., atoms, ions and radicals. Theoretically, photocatalysis is the conversion of light energy into chemical energy to produce radicals and other unstable chemical compounds. The primary oxidizing species formed during photocatalysis are hydroxyl radicals and superoxide anions [62]. The hexagonal wurtzite crystal form of pure ZnO is significantly used in many industrial and practical applications. Rakhshaei et al. reported that ZnO NPs with hexagonal phase show significant photocatalytic properties [63]. When a photon having energy greater than the band gap energy of ZnO strikes on its surface, electrons are released. The released electron further reacted with atmospheric oxygen to become a super oxide anion (O_2_^−^). The surface that has lost an electron takes another electron from moisture to fill up the hole. This converted the moisture into a hydroxyl radical (^•^OH). The two, i.e., the hydroxyl radical (^•^OH) and the superoxide anion (O_2_^−^) are highly reactive, and due to their strong oxidative power, they decompose organic compounds that cause staining. In our previous investigation, we explained the role of reactive oxygen species (ROS) during photocatalysis and reported that hydroxyl radicals (^•^OH) are the major radical scavengers that are responsible for the degradation of pollutants [56]. More production of ROS on the surface of the photocatalyst increases their power to degrade pollutants. The general mechanism of photocatalysis on the surface of nano ZnO is illustrated in Figure 9.

### 3.3. Modelling and Optimization

The experimental design (CCD) and response surface plots, i.e., surface plots and contour plots, were analysed to investigate the effects of input variables onto response. In modelling and optimisation, 3D surface plots and the 2D contour plot are helpful to visualise the effect of independent variables onto dependent variables. In total, 20 experimental samples were designed with variation in reagents amount and sonication time as illustrated in Table 2. For better visualisation of the drawn plots and for more accuracy regarding the analysis, Design-Expert 10 was used for statistical analysis. The results presented in Table 2 indicate that the photocatalytic activity of the developed samples increased with an increase in ZnCl_2_ and NaOH amount up to 15 g and 10 g, respectively. The results showed that MB removal (%) increased from 62.4% to 95.8%, 78.1% to 84.3% and 58.4% to 88.1%, by increasing active reagents amount and sonication time up to their maximum levels. However, the best outcome of 99.3% was achieved with optimal conditions, i.e., ZnCl_2_ 15 g, NaOH 10 g and sonication time 60 min, whereas, the predicted response value for MB removal (%) at optimal conditions (Sample 3) was 99.7%.

**Figure 10:**
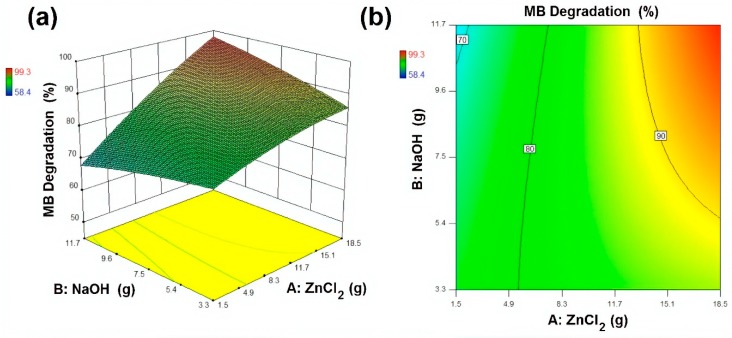
Response surface and contour plot for MB degradation (%) as a function of ZnCl_2_ and NaOH where (**a**) Response surface (**b**) contour plot respectively.

**Figure 11:**
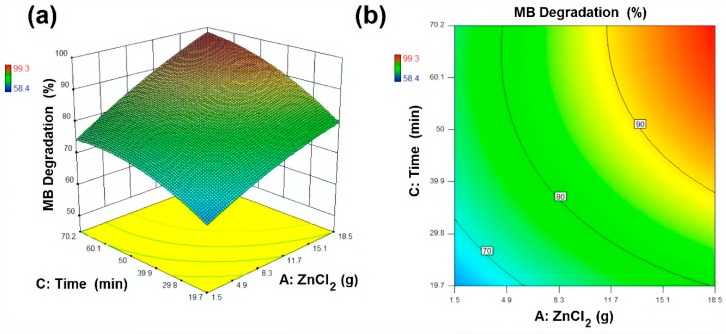
Response surface and contour plot for MB degradation (%) as a function of ZnCl_2_ and ultrasonic irradiation time where (**a**) Response surface (**b**) contour plot respectively.

**Figure 12:**
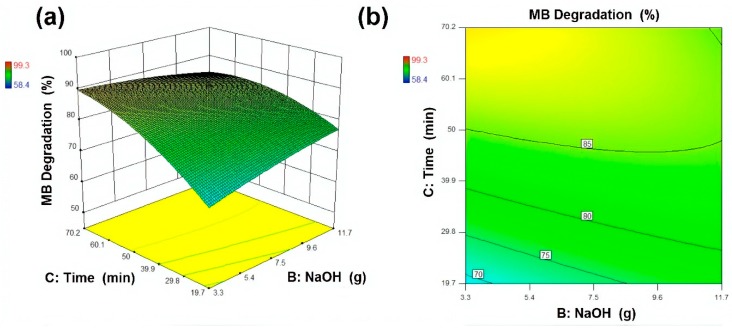
Response surface and contour plot for MB degradation (%) as a function of NaOH and ultrasonic irradiation time where (**a**) Response surface (**b**) contour plot respectively.

A mathematical model (Equation (4)) was suggested to evaluate the obtained results and the relationship between selected variables and response surfaces. The designed response by the model was a function of independent variables. This model is beneficial in the prediction of selected variables at any given point in space. By using this model, response surfaces and contour plots were drawn and analysed, as presented in Figure 10, Figure 11 and Figure 12. MB removal (%) on the basis of the designed model is calculated by the following Equation (4).

(4) $$\begin{array}{ll} MB\;Removal\;\left(\%\right) & \\ & =5.26-0.99\left(ZnCl_{2}\right)+2.56\left(NaOH\right)+2.36\left(Time\right)\\ & +0.40\left(ZnCl_{2}\times NaOH\right)+0.02\left(ZnCl_{2}\times Time\right)\\ & -0.09\left(NaOH\times Time\right)-0.06{\left(ZnCl_{2}\right)}^{2}-0.13{\left(EG\right)}^{2}-0.02{\left(Time\right)}^{2} \end{array}$$

Analysis of variance (ANOVA) was used to examine the interaction of data between selected variables and obtained response from samples 1–20. The results were further analysed to judge the goodness of fit. The results showed that the designed model for MB removal (%) is statistically significant for an F-value of 35.32 and prob > F of <0.0001, as shown in Table 4. R-squared coefficient was used to predict the fit of the model. The results explained that only 3.05% of the total variables cannot be explained by the designed model for MB removal (%). Low coefficient of variation (CV%) values of the developed model explained the precision and accuracy of the results and reliability of the experiment.

**Figure 13:**
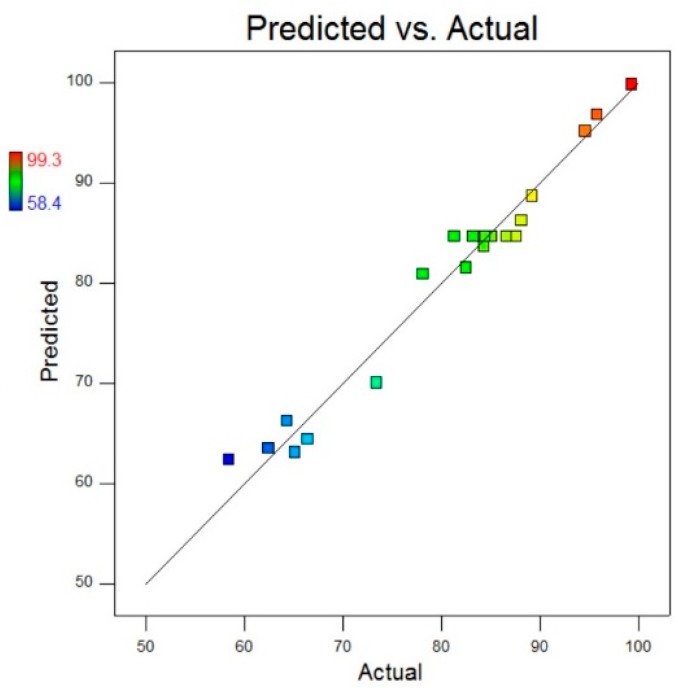
A plot of actual responses vs. predicted responses for MB removal (%).

**Figure 14:**
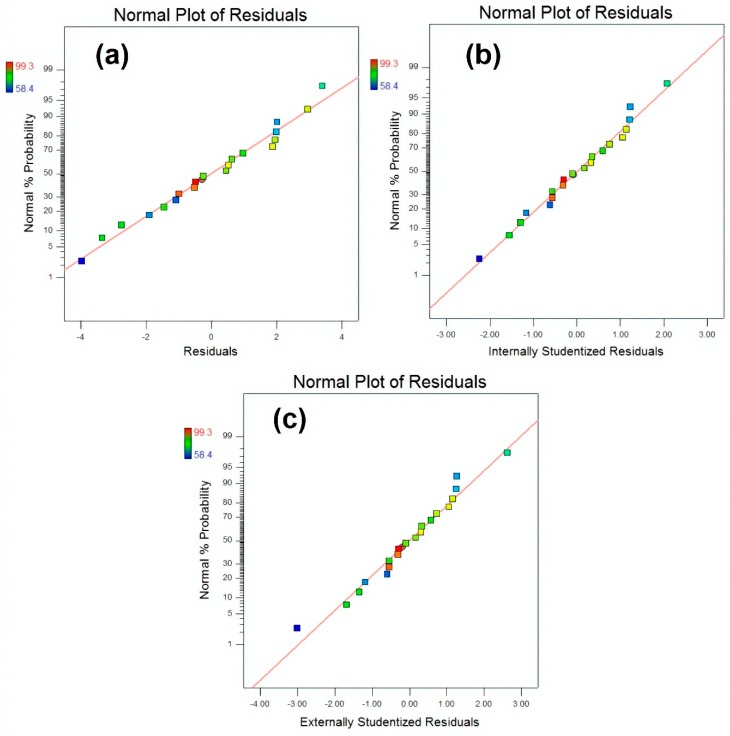
Normal plot of residuals for (**a**) raw residuals (**b**) internally studentized residuals and (**c**) externally studentized residuals respectively.

**Figure 15:**
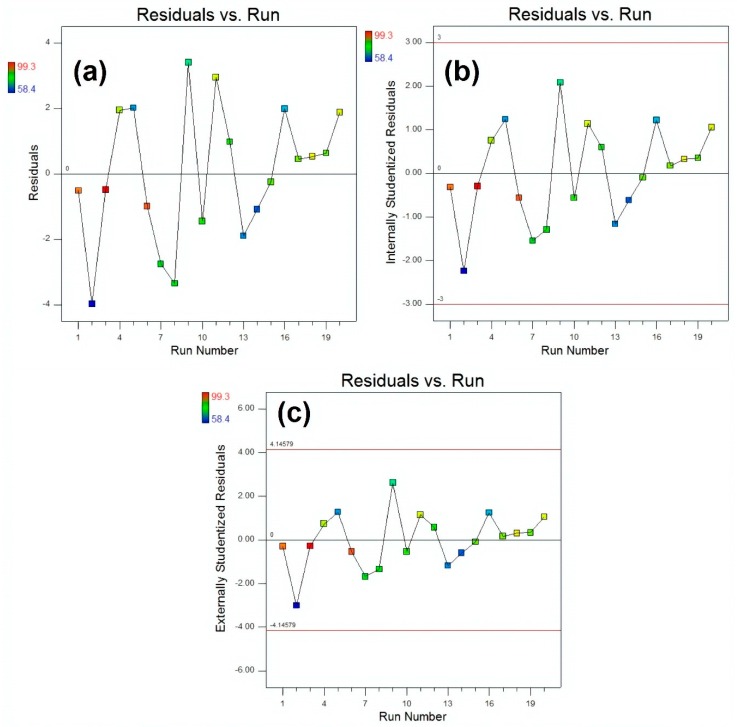
The plots of residuals vs. run for (**a**) raw residuals (**b**) internally studentized residuals and (**c**) externally studentized residuals respectively.

A comparison of actual values and predicted values for MB removal (%) is illustrated in Figure 13 that was used to detect the values that were not detected by the designed model. This comparative plot explains the overall pros of our fitted model in terms of statistical analysis and absolute residual minimisation. It was observed that the experimental values were very close to the normal distribution line, indicating a good fit of the model. The normal probability plot of residuals for raw residuals, internally studentised residuals and externally studentised residuals regarding MB degradation, is illustrated in Figure 14 that explained the relationship of experimental data with the standardised normal distribution, whereas the plot between residuals and experimental run order for raw residuals, internally studentised residuals and externally studentised residuals regarding MB degradation is presented in Figure 15, respectively. These graphs explained the lurking factors that influenced the response during experimentation. The optimal design points on the basis of Table 2 and Equation (4) are 15 g ZnCl_2_, 10 g NaOH and 60 min sonication time.

## 4. Reusability Performance of ZnO NPs for Sequential Applications

**Figure 16:**
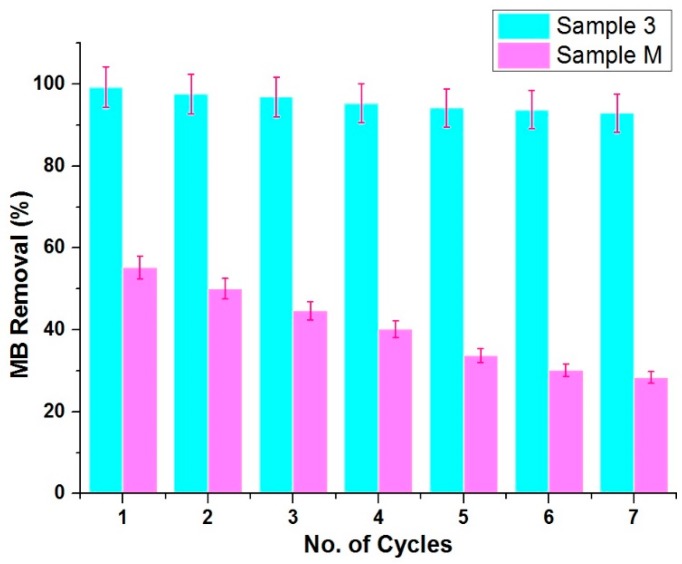
Reusability performance of sonochemical method (Sample 3) vs. conventional method (Sample M) as a photocatalysts against MB removal (%).

Reusability of ZnO NPs is an essential attribute from the point of sequential application. The reusability performance of Sample 3 (sonochemical method) and Sample M (conventional method) was estimated in the photocatalytic removal of MB for seven reuse cycles. After each cycle, both samples were extracted from their respective solutions by centrifuge process and washed with ethanol and then distilled water, respectively. After drying the samples at 100 °C for 1 h, they were reused again in the fresh MB solution until the seventh cycle. As shown in Figure 16, only a 6.1% MB degradation loss was observed for a sonochemically-prepared sample (Sample 3) even after seven reused cycles, while 26.9% loss was found for the conventional method (Sample M), respectively. Interestingly, the morphology was unchanged before and after each cycle. The overall results regarding the photodegradation and reusability confirmed that the sonochemical method is a facile and durable method for the synthesis of robust and more photocatalytically-active ZnO NPs than the conventional method.

## 5. Conclusions

ZnO NPs with pure hexagonal wurtzite crystalline structure were successfully synthesised by a one pot sonochemical method using ZnCl_2_ and NaOH as reactive variables. The samples prepared by the sonochemical method showed a more significantly enhanced photocatalytic performance than the conventional method (Sample M). The average particle size for ZnO NPs prepared by this sonochemical method was three times smaller than for that conventional method (Sample M). Ultrasonic irradiations played a remarkably crucial role in order to synthesise hexagonal wurtzite crystals of nano ZnO with smaller particle size and higher photocatalytic activity as compared to the conventional method. The photo degradation of MB dye recommends the potential use of sonochemically prepared ZnO NPs in waste water treatment for industries, especially the textile industry. Reusability of the prepared ZnO NPs during photocatalytic removal of the MB dye confirmed their durability for industrial applications. The as prepared nano ZnO could be further utilised in many other functional textile applications i.e., self-cleaning and UV protection.

## Figures and Tables

**Table 1:** The factors level setting of a 3-factors CCD matrix under coded values for the synthesis of zinc oxide nanoparticles (ZnO NPs).

Experimental Trial	Factors Level Setting		
	A	B	C
1	−1	−1	−1
2	1	−1	−1
3	−1	1	−1
4	1	1	−1
5	−1	−1	1
6	1	−1	1
7	−1	1	1
8	1	1	1
9	−α	0	0
10	α	0	0
11	0	−α	0
12	0	α	0
13	0	0	−α
14	0	0	α
15	0	0	0
16	0	0	0
17	0	0	0
18	0	0	0
19	0	0	0
20	0	0	0

**Table 2:** The 3-factors CCD matrix based on experimental values for actual variables and for experimental and predicted response of methylene blue (MB) removal.

Sample Number	ZnCl_2_ (g)	NaOH (g)	Sonication Time (min)	MB Removal (%) Experimental	MB Removal (%) Predicted
Sample M	15	10	−	55.3	−
1	15	5	60	94.6	95.1
2	10	7.5	19.7	58.4	62.3
3	15	10	60	99.3	99.7
4	10	7.5	45	86.6	84.6
5	5	10	30	65.1	63.0
6	18.5	7.5	45	95.8	96.7
7	10	3.3	45	78.1	80.8
8	10	7.5	45	81.3	84.6
9	15	5	30	73.4	70.0
10	10	7.5	45	83.2	84.6
11	10	7.5	45	87.6	84.6
12	5	5	60	82.5	81.5
13	5	10	60	64.3	66.2
14	1.5	7.5	45	62.4	63.4
15	10	7.5	45	84.4	84.6
16	5	5	30	66.4	64.4
17	10	7.5	45	85.1	84.6
18	15	10	30	89.2	88.6
19	10	11.7	45	84.3	83.6
20	10	7.5	70.2	88.1	86.2

**Table 3:** Results of specific surface area and pore volume distribution of ZnO NPs.

Sample No.	Surface Area (m^2^·g^−1^)	Pore Volume (cm^3^·g^−1^)	Pore Size (nm)
Sample M	53	0.51	49
1	104	0.30	23
2	105	0.27	21
3	111	0.20	16
4	108	0.25	19
5	107	0.27	19
6	105	0.28	22
7	106	0.24	21
8	108	0.23	19
9	105	0.26	22
10	106	0.25	21
11	107	0.24	20
12	109	0.21	19
13	108	0.22	19
14	106	0.23	21
15	107	0.26	20
16	108	0.22	19
17	106	0.24	22
18	109	0.20	18
19	107	0.21	19
20	108	0.23	18

**Table 4:** Analysis of variance (ANOVA) results regarding MB removal (%) for ZnO NPs.

Source	Sum of Squares	df	Mean Square	F Value	*p*-Value Prob > F	Remarks
Model	2560.20	9	284.47	35.32	<0.0001	Significant
A-ZnCl_2_	1322.18	1	1322.18	164.15	<0.0001	Significant
B-NaOH	9.55	1	9.55	1.19	0.3017	Not significant
C-Sonication Time	680.84	1	680.84	84.53	<0.0001	Significant
AB	200.00	1	200.00	24.83	0.0006	Significant
AC	32.00	1	32.00	3.97	0.0742	Not significant
BC	98.00	1	98.00	12.17	0.0058	Significant
A^2^	36.34	1	36.34	4.51	0.0596	Not significant
B^2^	10.26	1	10.26	1.27	0.2855	Not significant
C^2^	191.87	1	191.87	23.82	0.0006	Significant
Residual	80.55	10	8.05	-	-	-
Lack of Fit	54.47	5	10.89	2.09	0.2191	Not significant
Pure Error	26.08	5	5.22	-	-	-
Cor Total	2640.75	19		-	-	-

R-squared: 0.9695, adjusted R-squared: 0.9420, CV%: 3.53.
